# Multi-Omics Analysis of Gut Microbiota and Sperm Quality in Tibetan Breeding Boars

**DOI:** 10.3390/metabo15070447

**Published:** 2025-07-02

**Authors:** Mingxuan Zhao, Mengjia Han, Hongliang Zhang, Xiangdong Wang, Yikai Yin, Jian Zhang, Peng Shang

**Affiliations:** 1 College of Animal Science, Xizang Agriculture and Animal Husbandry University, Nyingchi 860000, China; m19863998873@163.com (M.Z.); 15515762043@163.com (M.H.); holiangzhang@126.com (H.Z.); 13993473560@163.com (X.W.); yyk0426@126.com (Y.Y.); 2 Key Laboratory of Tibetan Pig Genetic Improvement and Reproduction Engineering, Nyingchi 860000, China; 3 Tibetan Pig Science and Technology Courtyard in Nyingchi, Nyingchi 860000, China

**Keywords:** gut microbiota, gut–testis axis, metabolomics, sperm quality, 16S rRNA

## Abstract

Background/Objectives: Reproductive efficiency in breeding boars critically impacts swine industry productivity, with sperm quality being multifactorially regulated by gut microbiota. This study aimed to elucidate the microbiota–metabolite interactions underlying sperm quality differences in Tibetan boars. Methods: Integrated 16S rRNA sequencing and untargeted metabolomics were performed on fecal and semen samples from eight healthy Tibetan boars (31–33 months old), stratified into low-semen (CJ) and high-semen utilization (HJ) groups. Analyses included sperm quality assessment, microbial profiling, and metabolic pathway enrichment. Results: The HJ group exhibited significantly enhanced sperm motility and semen utilization rates (*p* < 0.05). Gut microbiota composition differed markedly, with Firmicutes and Proteobacteria enriched in HJ boars. Metabolomics identified key metabolites positively correlated with sperm quality (e.g., butyrate, phenyllactic acid), while lithocholic acid showed negative associations. KEGG analysis revealed predominant involvement in butanoate metabolism and bile acid biosynthesis. Core microbiota (e.g., Ruminococcus) modulated sperm quality through short-chain fatty acid networks and bile acid homeostasis. Conclusions: Gut microbiota regulated the sperm microenvironment via a “metabolic-immune” dual pathway mediated by the gut–testis axis. These findings establish a theoretical basis for probiotic or metabolite-targeted strategies to improve boar reproductive performance.

## 1. Introduction

The semen quality of Tibetan boars is highly susceptible to environmental stressors, particularly the prevalent hypoxic conditions in plateau regions, which significantly impair sperm quality and reduce semen preservation duration [1,2]. As genetic superiority in livestock forms the cornerstone of modern animal husbandry and a key focus in agricultural biotechnology, optimal semen resources are critical for maximizing breeding efficiency [3,4,5]. Semen quality not only reflects the genetic potential of male animals but also serves as a pivotal determinant of reproductive success, making it an essential criterion for genetic selection and the realization of breeding potentia [6,7]. Sperm quality, a core metric for assessing reproductive performance, is regulated by multifaceted factors including genetics, nutritional status, environmental influences, and gut microbiota dynamics [8,9].

The gut microbiota, often termed the “second genome,” comprises trillions of co-evolved bacteria, viruses, and fungi that function together as an endocrine organ, secreting metabolites into systemic circulation to modulate host physiology [10,11]. In livestock, gut microbes are categorized into beneficial (e.g., *Bifidobacterium*, *Lactobacillus*), pathogenic (e.g., *Staphylococcus*, *Salmonella choleraesuis*, *Klebsiella*), and neutral species (e.g., *Escherichia coli*, *Enterococcus faecalis*), which collectively influence intestinal motility, pathogen resistance, and metabolic homeostasis [12,13]. Spatial heterogeneity exists within the gut ecosystem: lactic acid bacteria dominate the stomach and small intestine, while obligate anaerobes (e.g., Bacteroidaceae, Prevotellaceae, Rikenellaceae) prevail in the colon [14,15]. Emerging evidence highlights the gut–testis axis as a novel pathway through which gut microbiota modulates testicular microenvironments and spermatogenesis [16,17]. Microbial-derived metabolites, such as short-chain fatty acids (SCFAs), and immune-regulatory pathways have been implicated in energy metabolism and reproductive function [18,19,20]. Notably, high-fat diet-induced dysbiosis in murine models suppresses spermatogenesis and reduces sperm motility, underscoring the urgency to elucidate microbiota-mediated mechanisms affecting male fertility [21,22].

Gut microbial composition is shaped by host genetics, sex, age, and environmental factors [23]. Host-microbiota co-adaptation enables bacteria to synthesize metabolites that maintain microbial equilibrium [24,25]. Sex-specific differences are evident, with males exhibiting higher abundances of Bacteroides and Prevotella compared to females. Recent studies emphasize bidirectional interaction between gut microbiota and reproductive health. For instance, fecal microbiota transplantation from high-fat diet-fed mice to healthy counterparts elevated Bacteroides and Prevotella abundance, concomitant with diminished sperm quality [21]. Similarly, glyphosate exposure in mice disrupted gut microbiota (e.g., altered Bacteroidetes/Firmicutes ratios) and induced testicular dysfunction, with *Prevotella* and *Bacteroides* abundance inversely correlating with sperm parameters [26]. In sheep models, metabolic disorder-induced depletion of Ruminococcaceae-NK4A214-group impaired vitamin A absorption, leading to defective spermatogenesis via systemic circulation [27]. Conversely, probiotic-enriched fermented feed enhanced mouse semen quality, elevating sperm motility and progressive movement (A-grade sperm), likely through microbial modulation [28]. Despite these advances, prior studies relying solely on 16S rRNA sequencing lack functional insights into the microbiota–host crosstalk.

Integrative multi-omics approaches offer unprecedented opportunities to decode microbiota–reproductive interactions [29]. While 16S rRNA sequencing tracks compositional shifts, metabolomics identifies host–microbe co-metabolites (e.g., bile acids, tryptophan derivatives) that mediate physiological outcomes [30]. For example, microbial indole derivatives regulate oxidative stress during spermatogenesis via tryptophan metabolic pathways [31,32,33]. However, gaps persist in understanding how gut microbial metabolic networks govern germ cell development via the gut–testis axis in boars. This study employed 16S rRNA sequencing and metabolomics to delineate gut microbiota signatures in boars with divergent semen quality, identified reproductive performance-linked microbial biomarkers and metabolic pathways, and unraveled their mechanistic roles in spermatogenesis. Our findings will inform precision nutrition strategies for boars and advance microbiota-targeted interventions to enhance livestock fertility.

## 2. Methods

### 2.1. Breeding Boars and Sample Collection

Eight healthy Tibetan breeding boars (31–33 months old) were randomly selected from the Tibetan swine breeding base in Zengba Village, Linzhi City. All animals were fed a standardized commercial formula diet (Yunmu, Linzhi, China) (see Table 1 for composition). All sample collection procedures were performed consistently by the same trained operators. During semen collection, these operators wore sterile surgical gloves to ensure sample purity (ABclonal, Wuhan, China), collecting 2 µL of semen suspension. Fresh fecal samples were simultaneously collected using sterile 5 mL cryovials (ABclonal, Wuhan, China). All fecal specimens were placed on ice immediately after collection and subsequently stored at −80 °C for downstream analysis.

The premix supplied the following per kilogram of concentrate: vitamin A (10,000 IU), vitamin D3 (550 IU), vitamin E (20 IU), biotin (0.06 mg), Cu (22 mg as sulfate), Fe (94 mg as sulfate), Mn (80 mg as sulfate), Zn (88 mg as sulfate), I (0.75 mg as potassium iodide), Se (0.50 mg as sodium selenite), Ca (0.35%), P (0.125%), and NaCl (0.80%).

### 2.2. Assessment of Sperm Motility and Quality

A 20 microliter aliquot of the semen suspension was aspirated using a micropipette (ABclonal, Wuhan, China), dropped onto a glass slide (HaiBo Biotechnology, Qingdao, China), and covered with a cover slip (HaiBo Biotechnology, Qingdao, China). The glass slide was placed on the microscope stage (Yuantian Aori, Beijing, China), and the sample temperature was maintained at 37 °C using a heating stage to ensure natural sperm motility. A Computer Aided Sperm Analysis (CASA) system (Yuantian Aori, Beijing, China) was initiated. The sample was observed under a microscope, the trajectory of sperm movement was captured, and the data were recorded [34]. All procedures strictly adhered to aseptic protocols to ensure experimental accuracy and reliability.

Based on semen utilization rates, four boars with values below 60% were categorized as the low-semen utilization group (CJ group), while another four boars exhibiting 80% semen utilization were designated as the high-semen utilization group (HJ group).

Sperm motility evaluation: The CASA system (computer-assisted sperm analysis system) was employed to quantify kinetic parameters, including total motility (%), progressive motility (PR%), and motion characteristics (e.g., curvilinear velocity, straight-line velocity). High-speed videomicroscopy (200 frames/s) captured sperm trajectories, with data processed using integrated software (CASA v3.4) following WHO guidelines [35].

### 2.3. Fecal Sample Pretreatment

Freeze-dried feces were pulverized (60 Hz, 30 s) and 50 mg aliquots were weighed into centrifuge tubes (ABclonal, Wuhan, China). A 700 μL volume of extraction solvent (Kelong, Beijing, China) (methanol/water, 3:1 *v*/*v*, pre-cooled to −40 °C with internal standards) was added, followed by vortex-mixing (30 s), homogenization (35 Hz, 4 min), and ice-water bath sonication (Yuantian Aori, Beijing, China) (5 min). The homogenization–sonication cycle was repeated thrice, after which samples were incubated overnight at 4 °C on a rotary mixer (Yuantian Aori, Beijing, China).

Samples were centrifuged at 12,000× *g* (13,800× *g*, radius 8.6 cm) for 15 min at 4 °C. Supernatants were carefully filtered through 0.22 μm membranes and diluted fivefold with extraction solvent. After vortexing (30 s), 40 μL aliquots from each sample were pooled to generate QC samples, which were stored at −80 °C until instrumental analysis [36].

### 2.4. Microbial Community Sequencing of Breeding Boar Feces

Genomic DNA was extracted from fecal samples, followed by amplification of the rDNA conserved regions using barcode-indexed primers (Table 2). PCR products were gel-purified, quantified via QuantiFluorTM fluorometer, and pooled in equimolar ratios. Sequencing adapters were ligated to construct libraries, which were sequenced on an Illumina PE250 platform [37].

Raw sequencing reads were processed using the DADA2 pipeline for quality filtering, denoising, and generation of ASVs, equivalent to 100% similarity-clustered OTUs. The DADA2 algorithm merged paired-end reads via overlap alignment, performed chimera removal, and yielded high-quality clean data.

Post-ASV/OTU generation, bioinformatics analyses included taxonomic annotation, α-diversity and β-diversity assessments, and functional prediction of microbial communities. Differential abundance testing and comparative analyses were conducted between predefined groups (CJ vs. HJ) using validated statistical frameworks.

### 2.5. LC-MS Analysis

Chromatographic separation was performed using an EXIONLC System (ExionLC™ 2.0+, SCIEX, Toronto, ON, Canada) coupled with a Waters UPLC column (Waters Corporation, Milford, MA, USA). Mass spectrometric detection was conducted on a SCIEX 6500 QTRAP+ triple quadrupole mass spectrometer equipped (SCIEX, Toronto, ON, Canada) with an IonDriveTurbo V ESI source in multiple reaction monitoring (MRM) mode. Ion source parameters were set as follows: IonSpray Voltage: +5500/−4500 V, Curtain Gas: 35 psi, Temperature: 400 °C, Ion Source Gas 1: 60 psi, Ion Source Gas 2: 60 psi, and DP: ±100 V. Raw data acquisition and quantitative analysis were executed via SCIEX Analyst WorkStation Software (Version 1.6.3, Toronto, ON, Canada). MS raw files were converted to TXT format using MSconverter software (ProteoWizard v3.0.22106, ProteoWizard Foundation, Palo Alto, CA, USA), followed by peak extraction and annotation with an in-house R package v2.3.1 and a customized metabolite database [38].

### 2.6. Data Analysis

Raw data were processed using Compound Discoverer v3.3 software (Compound Discoverer 3.3) for peak alignment, identification, and metabolite quantification. Statistical analysis included a Student’s *t*-test performed in SPSS v21.0 software and Spearman correlation analysis between gut microbial relative abundance and fecal metabolites using a GraphPad Prism v8.0. Data are expressed as mean ± standard error, with statistical significance defined at (*p* < 0.05).

## 3. Results

### 3.1. Semen Parameters of Breeding Boars

As indicated in Table 3, no significant difference in sperm density was observed between the low semen utilization group (CJ group) and the high semen utilization group (HJ group). However, the HJ group exhibited significantly elevated values in key semen quality parameters, including semen utilization rate, total motility, and progressive motility, compared to the CJ group (*p* < 0.05).

### 3.2. Gut Microbial Community Composition

**Figure 1:**
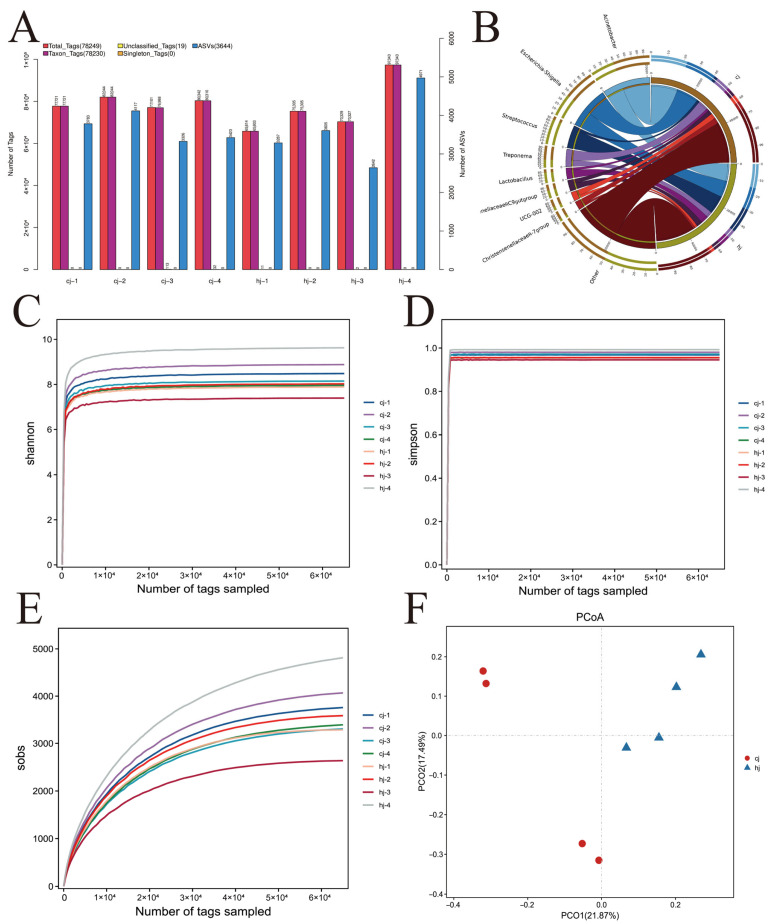
Gut microbiota diversity and biomarker analysis in breeding boars with divergent semen quality (**A**) Venn diagram illustrating amplicon sequence variant (ASV) distribution between the high semen utilization group (HJ group) and low semen utilization group (CJ group); (**B**) Circos plot of core taxa (top 10 relative abundance at phylum to genus levels, ASV ≥ 2000) across groups; (**C**–**F**) Alpha diversity indices (Shannon, Simpson, observed OTUs) and beta diversity (weighted UniFrac PCoA).

To investigate differences in gut microbiota composition between boars with divergent semen quality, fecal samples from eight individuals were analyzed via 16S rRNA gene sequencing, yielding 625,999 high-quality sequences (range: 65,814–97,343 per sample). Clustering identified 5087 amplicon sequence variants (ASVs), with 1726 ASVs unique to the high-semen quality group (HJ group), 2038 ASVs unique to the low-semen quality group (CJ group), and 1323 shared ASVs, indicating both divergence and conserved core microbiota features between groups (Figure 1A).

The top 10 taxa (phylum to genus) with relative abundance ≥ 0.1% and ASV counts ≥ 2000 were visualized using circos plots to illustrate cross-group distribution patterns (Figure 1B). Alpha diversity (observed OTUs, Shannon, Simpson indices) and beta diversity (weighted UniFrac-based PCoA) analyses revealed no significant intergroup differences in species richness or diversity (independent-samples *t*-test, *p* > 0.05) (Figure 1C–F).

**Figure 2:**
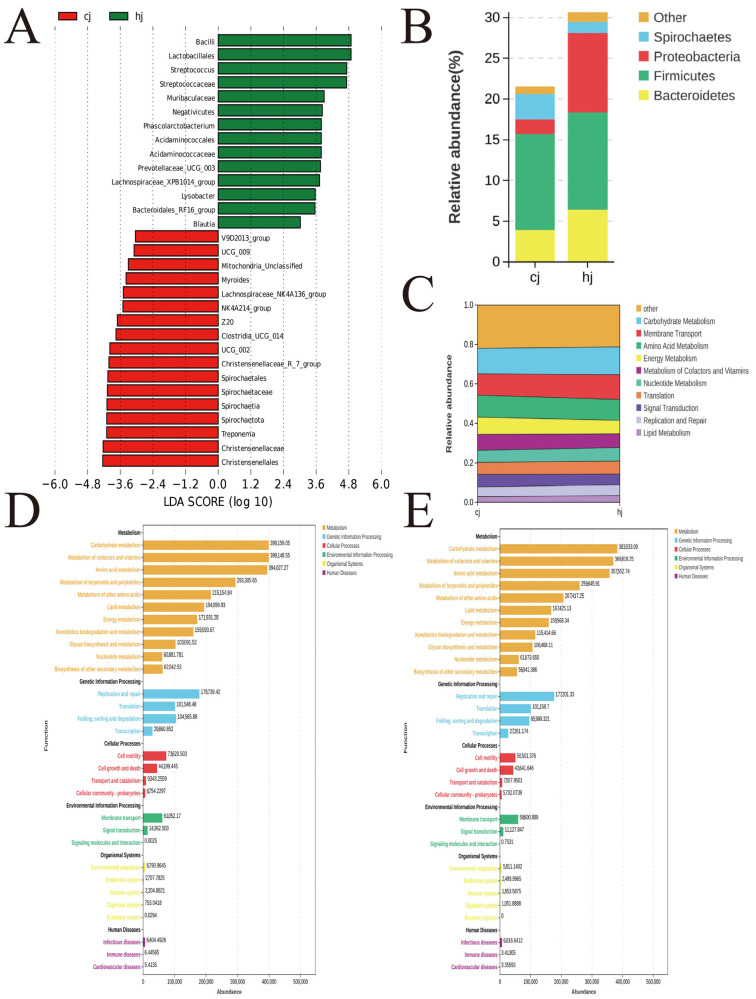
Gut microbial composition and functional pathway divergence; (**A**) LEfSe analysis highlighting discriminant taxa (LDA score > 3.0); (**B**) Phylum-level abundance differences and microbial community separation (weighted UniFrac PCoA); (**C**) Sankey diagram mapping PICRUSt-predicted metabolic flux variations; (**D**,**E**) KEGG pathway enrichment (level 3) based on PICRUSt prediction for HJ and CJ groups.

Linear discriminant analysis effect size (LEfSe) identified semen quality-associated microbial biomarkers (Figure 2A). Multilevel analysis involved Kruskal–Wallis tests for preliminary screening, followed by pairwise Wilcoxon tests and linear discriminant analysis (LDA) to calculate effect sizes. The left panel of Figure 1C displays LDA scores (LDA score; log-transformed), reflecting taxon contribution to group separation, while the right panel presents a cladogram mapping differential taxa across taxonomic hierarchies (phylum to genus). Node sizes correlate with relative abundance, and colors denote group specificity. Key discriminative taxa for the HJ group included *Bacillus*, *Lactobacillus*, *Streptococcus*, Streptococcaceae, and Lachnospiraceae.

However, principal coordinates analysis (PCoA) demonstrated significant separation in the microbial community structure between HJ and CJ groups. Statistical analysis employed independent-samples *t*-tests for parametric data and Wilcoxon rank-sum tests for nonparametric data, with significance at (*p* < 0.05). Dominant phyla across both groups included Firmicutes, Bacteroidota, Spirochaetota, Proteobacteria, and Actinobacteriota, collectively accounting for >95% of total microbiota. The HJ group exhibited significantly higher relative abundances of Firmicutes and Proteobacteria, alongside elevated Spirochaetota levels compared to the CJ group (Figure 2B,C).

### 3.3. Functional Prediction of Gut Microbiota

Functional profiling of gut microbiota was performed using PICRUSt (Phylogenetic Investigation of Communities by Reconstruction of Unobserved States) based on 16S rRNA sequences, with functional annotation via the KEGG database. Comparative analysis (Student’s *t*-test) revealed six differentially abundant KEGG level-3 pathways between the high semen utilization group (HJ group) and low semen utilization group (CJ group) (Figure 2D,E). The HJ group exhibited distinct functional enrichments in metabolism, genetic information processing, cellular processes, environmental adaptation, and human disease-related pathways, suggesting microbiota-mediated regulation of sperm quality through energy metabolism optimization and immune modulation.

Notably, the HJ group showed significant enrichment (*p* < 0.05, independent-samples *t*-test) in carbohydrate metabolism, cofactor and vitamin metabolism, lipid metabolism, and xenobiotics biodegradation pathways. These metabolic advantages may enhance semen quality through the following mechanisms.

### 3.4. Fecal Metabolite Profiles in Breeding Boars

**Figure 3:**
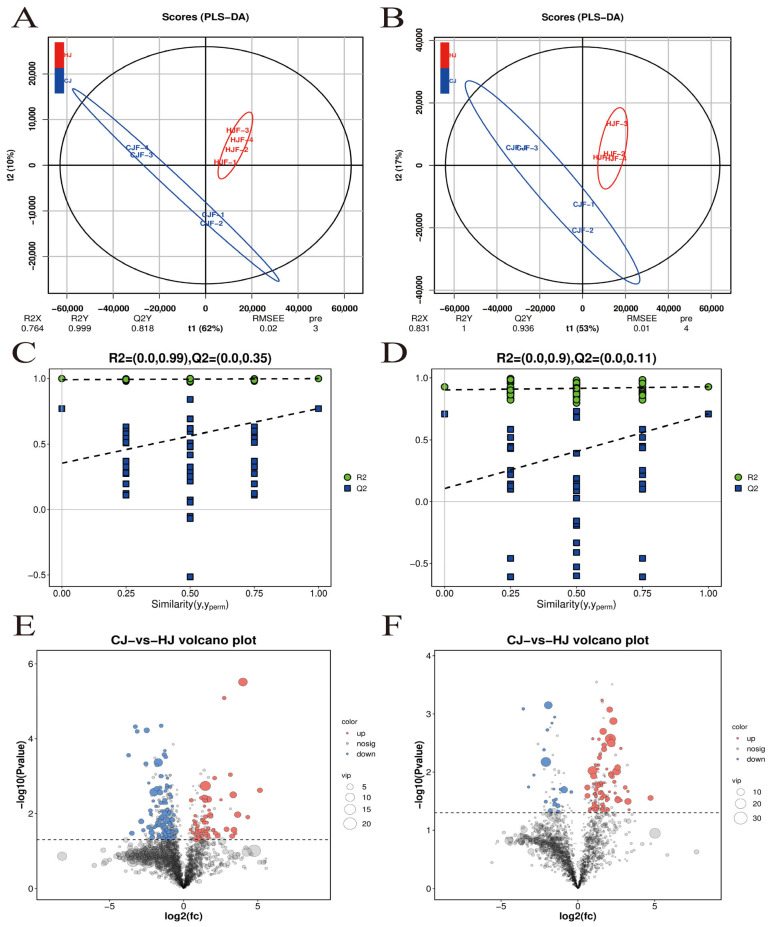
Classification model analysis of high and low semen utilization groups based on untargeted metabolomics (**A**) PLS-DA score plot (positive ion mode); (**B**) PLS-DA score plot (negative ion mode); (**C**) OPLS-DA model (positive ion mode); (**D**) OPLS-DA model (negative ion mode); (**E**) Volcano plot of differential metabolites (positive ion mode); (**F**) Volcano plot of differential metabolites (negative ion mode).

To investigate gut microbiota-derived metabolic differences between the low semen utilization group (CJ group) and high semen utilization group (HJ group), untargeted metabolomics was employed for qualitative and quantitative analysis of fecal metabolites. A total of 24,712 metabolites were identified across positive ion (POS) and negative ion (NEG) modes (POS: 13,325; NEG: 11,387). Associations between metabolite profiles and group classification were established using partial least squares regression (PLS-DA) and orthogonal partial least squares-discriminant analysis (OPLS-DA) models (Figure 3A–D). The models exhibited high stability and reliability (R^2^ = 0.99 for POS, R^2^ = 0.90 for NEG) without overfitting.

**Figure 4:**
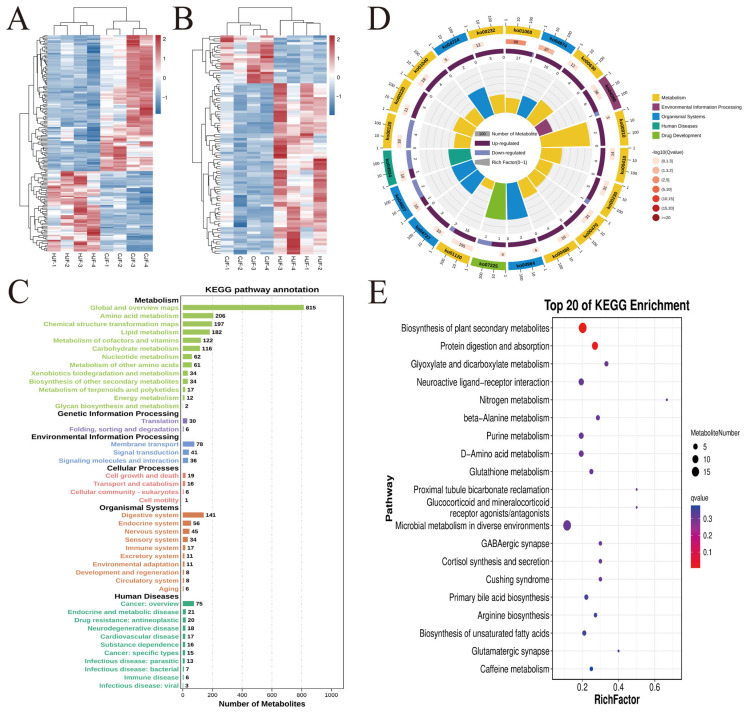
Differential metabolites and metabolic pathway analysis between high and low semen utilization groups (**A**) Clustering heatmap of differential metabolites (positive ion mode); (**B**) Clustering heatmap of differential metabolites (negative ion mode); (**C**) KEGG pathway classification histogram; (**D**) KEGG enrichment circle plot; (**E**) KEGG enrichment bubble plot.

Through OPLS-DA combined with independent-samples *t*-tests (*p* < 0.05, FDR correction), 263 significantly differential metabolites were identified: 167 in POS mode (62 upregulated, 105 downregulated) and 96 in NEG mode (75 upregulated, 21 downregulated) (Figure 3E,F). Key discriminant metabolites included 6-Hydroxyhexanoate, Lithocholic acid (LCA), Phenyllactic acid (PLA), Butanoic acid, Valeric acid, Pentadecanoic acid, Corticosterone, Mevalonic acid, and 4-Pyridoxic acid (Figure 4A,B). Semen utilization rates positively correlated with Butanoic acid, Valeric acid, Pentadecanoic acid, PLA, 6-Hydroxyhexanoate, Mevalonic acid, and 4-Pyridoxic acid, but negatively correlated with LCA and Corticosterone.

Enriched KEGG pathways included Biosynthesis of plant secondary metabolites, Protein digestion and absorption, Glyoxylate and dicarboxylate metabolism, Neuroactive ligand-receptor interaction, Nitrogen metabolism, Beta-alanine metabolism, Purine metabolism, D-Amino acid metabolism, Glutathione metabolism, Proximal tubule bicarbonate reclamation, Glucocorticoid and mineralocorticoid receptor agonists/antagonists, Microbial metabolism in diverse environments, GABAergic synapse, Cortisol synthesis and secretion, Cushing syndrome, Primary bile acid biosynthesis, Arginine biosynthesis, Biosynthesis of unsaturated fatty acids, Glutamatergic synapse, and Caffeine metabolism (Figure 4C–E).

### 3.5. Microbiota-Metabolite Correlation Analysis

**Figure 5:**
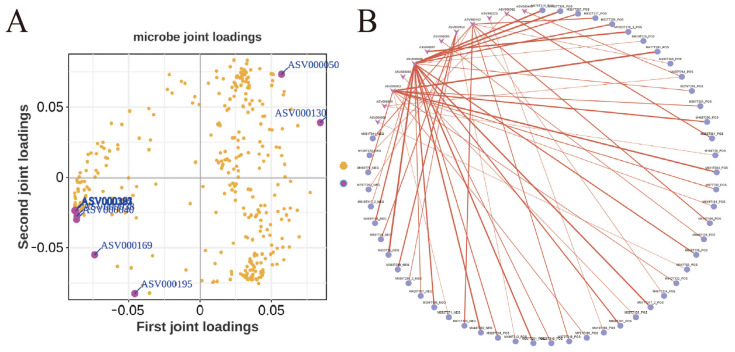
Correlation between differential metabolites and semen quality-associated microbiota (**A**) Microbial loading plot of differential metabolites associated with semen quality; (**B**) Correlation network of differential metabolites and semen quality-associated microbiota, note:red lines represent statistically significant correlations (*p* < 0.05).

Integrating 16S rRNA sequencing data (genus-level differential taxa) and untargeted metabolomics results (differential metabolites), Pearson’s correlation coefficients were calculated to assess associations between microbial diversity and metabolite profiles, visualized via correlograms (Figure 5A,B). Multi-omics integration revealed potential functional links between unclassified microbial taxa and metabolite dynamics.

*Ruminococcus* exhibited significant positive correlations with short-chain fatty acids (SCFAs) (e.g., butyrate, valerate) and phenyllactic acid (PLA), suggesting its role in enhancing cellulose degradation and anti-inflammatory metabolite synthesis, thereby improving intestinal energy supply and mitigating sperm oxidative stress. Conversely, *Eubacterium_siraeum_group* and *Romboutsia* negatively correlated with secondary bile acids (e.g., lithocholic acid, LCA), likely suppressing LCA production via 7α-dehydroxylase activity to maintain sperm membrane stability. Additionally, Rikenellaceae_RC9_gut_group and Christensenellaceae_R-7_group showed strong associations with branched-chain amino acids (BCAAs) and 4-pyridoxic acid (a vitamin B6 metabolite), implicating their regulatory roles in nitrogen metabolism and antioxidant pathways to influence semen utilization.

KEGG pathway enrichment further demonstrated that differential microbiota predominantly participated in butanoate metabolism (ko00650), primary bile acid biosynthesis (ko00120), and vitamin B6 metabolism (ko00750). These findings indicate that microbiota–metabolite interaction networks modulate boar reproductive health through multidimensional mechanisms involving nutrient provisioning, inflammatory regulation, and redox balance (Figure 5A,B).

## 4. Discussion

Semen quality is a critical indicator for evaluating the reproductive capacity of boars, with significant implications for the economic efficiency of the livestock industry [39,40]. In this study, Tibetan boars were categorized into high-semen quality (HJ group) and low-semen quality (CJ group) groups based on semen utilization rate, sperm motility, density, and progressive movement. Fecal metabolites, which are closely associated with gut microbiota, provided comprehensive metabolic insights [41,42,43]. We found that the gut microbial diversity in the low-semen quality group (CJ group) was lower than that in the high-semen quality group (HJ group), with significant differences in the relative abundance of multiple genera at the taxonomic level. At the phylum level, Firmicutes and Bacteroidota dominated the fecal microbiota in both groups. Simpson diversity curve analysis indicated that the Simpson index stabilized as the sequencing depth increased, with an average sequencing depth of approximately 78,249 tags per sample, sufficiently capturing microbial community evenness. Intergroup comparisons revealed that the HJ group exhibited a significantly lower Simpson index than the CJ group, indicating higher microbial community evenness and more balanced species distribution in the HJ group. This difference in evenness suggested that although both groups had similar microbial species richness, the HJ group’s microbiota structure was more stable, potentially supporting host physiological functions through synergistic interactions, such as suppressing the overgrowth of harmful bacteria or enhancing the metabolic activity of beneficial bacteria. Combined analysis of Sobs and Simpson diversity indices demonstrated no significant difference in species richness between the HJ and CJ groups, but the HJ group showed significantly higher microbial evenness. These findings align with previous studies showing no significant Shannon diversity index differences, further confirming that the core distinction lies in microbial distribution uniformity rather than species quantity [44]. Thus, microbial community evenness, rather than species richness, may be the key factor influencing semen quality.

In this study, *Bacilli*, *Streptococcus*, Lactobacillales, and Streptococcaceae were significantly enriched in the HJ group, while Muribaculaceae and Negativicutes were more abundant in the CJ group. These taxa may influence sperm quality by regulating host metabolism or inflammatory responses [45,46]. Through positive ion mode (POS) detection, 6-hydroxyhexanoate was identified as a metabolite with a significantly higher concentration in the HJ group (high semen utilization group), compared to the CJ group (low utilization group). This metabolite, verified via the MassBank database (ID: PR100501), was linked to “lipid metabolism” and “xenobiotic degradation” pathways. These results suggest that specific microbial taxa may regulate the spermatogenic microenvironment through dual “metabolic-immune” pathways, thereby influencing semen quality parameters.

Functional prediction and metabolic pathway analysis indicated that 6-hydroxyhexanoate may improve semen quality through three mechanisms: (1) Energy metabolism regulation: As an intermediate product of fatty acid β-oxidation, it enhances ATP supply via mitochondrial acetyl-CoA generation, supporting sperm motility [47]; (2) Xenobiotic detoxification: It participates in the hydroxylation of polycyclic aromatic hydrocarbons (PAHs) or plasticizers through the CYP450 enzyme system, converting them into water-soluble metabolites for excretion and reducing testicular oxidative damage [27]; (3) Membrane stability enhancement: Hydroxy fatty acids may stabilize the sperm membrane structure by modulating sphingomyelin synthesis, thereby reducing lipid peroxidation [48,49]. Lithocholic acid (LCA), a characteristic metabolite in the HJ group, improved sperm quality through synergistic “antioxidant-anti-inflammatory-energy metabolism” pathways. Its enrichment was closely associated with specific microbial taxa (e.g., Clostridiumsensu stricto 3), providing a theoretical basis for targeting gut microbiota to regulate bile acid metabolism and enhance boar reproductive performance. Further studies involving fecal microbiota transplantation or LCA intervention experiments are needed to validate these causal relationships. Phenyllactic acid (PLA) was positively correlated with the semen utilization rate, suggesting a potential link between its abundance and sperm quality. Its enrichment was linked to *Lactobacillus* activity, supporting the potential of probiotic interventions (e.g., *Lactobacillus* supplementation) to optimize boar reproductive performance [50]. Subsequent in vitro or animal studies are required to confirm PLA’s direct effects and dose dependency.

Butyrate, a core metabolite in the HJ group, showed a positive correlation with the semen utilization rate, suggesting a potential link between its abundance and sperm quality. Its enrichment was closely associated with symbiotic bacteria such as *Faecalibacterium prausnitzii*, supporting strategies to enhance boar nutrition through butyrate precursors (e.g., dietary fiber) or probiotics [51,52]. Studies have shown that host–gut microbiota interactions regulate semen quality via metabolites like butyrate, amino acids, vitamins, and bile acids [53]. The enrichment of *Bacilli*, *Lactobacillales*, *Streptococcus*, and Streptococcaceae in the HJ group likely enhanced host energy metabolism by promoting short-chain fatty acid (e.g., butyrate) synthesis, thereby improving sperm motility. When complex carbohydrates are ingested, gut microbiota produce substantial butyrate, which serves as the primary energy source for colonic cells, blocks NF-κB signaling to alleviate intestinal inflammation, and indirectly enhances testicular antioxidant capacity and testosterone (T) secretion, improving semen quality [54]. For instance, Al-Asmakh et al. demonstrated that butyrate from *Clostridium butyricum* modulates the blood–testis barrier permeability, stimulates gene expression in Leydig cells, and increases serum FSH, LH, and intratesticular T levels, promoting spermatogenesis [55]. Direct dietary supplementation with butyrate (e.g., 500 mg/kg sodium butyrate in adult roosters) elevates serum and testicular interstitial cell counts, increases sperm concentration, and reduces abnormal sperm rates [56]. Gubara et al. confirmed that 0.05% sodium butyrate supplementation in rooster diets improves testicular antioxidant levels, serum T secretion, semen volume, sperm density, and motility while reducing deformity rates [57]. Collectively, these findings indicate that butyrate enhances semen quality by elevating T levels. Therefore, dietary butyrate supplementation can directly target testicular function or indirectly improve semen quality by increasing gut *Clostridium*, *Enterobacter*, and *Coprococcusa* bundance to boost butyrate production [58]. Studies have validated that butyrate reduces spermatogenic cell apoptosis via AMPK pathway activation and suppresses testicular inflammation by inhibiting the TLR4/NF-κB pathway in murine models. Muribaculaceae and Negativicutesenrichment in the CJ group may trigger testicular inflammation via LPS-mediated TLR4/NF-κB activation, consistent with microbiota dysbiosis-induced spermatogenic defects in mice [55]. Follow-up studies should verify these causal effects through fecal microbiota transplantation or butyrate gavage experiments.

By integrating gut microbiota and metabolomic data, this study revealed potential associations between semen utilization differences and specific microbiota–metabolite interaction networks. Phenyllactic acid (PLA) may improve the seminal microbial environment by inhibiting reproductive tract pathogens (e.g., *Escherichia coli*) [58]. Results show that *Ruminococcus* was positively correlated with short-chain fatty acids (butyrate/valerate) and PLA, aligning with its role in cellulose degradation and acetate/butyrate synthesis pathways to enhance intestinal energy metabolism [59]. Short-chain fatty acids may maintain spermatogenic microenvironment homeostasis by activating GPR41 receptors to suppress testicular inflammation [60]. In this study, negative correlations between *Eubacterium_siraeum_group*/*Romboutsia* and lithocholic acid (LCA) suggest that these taxa may reduce oxidative damage to sperm membrane lipids by regulating secondary bile acid conversion via 7α-dehydroxylase activity, similar to human gut microbiota’s role in bile acid metabolism. Additionally, associations between Rikenellaceae_RC9_gut_group/Christensenellaceae_R-7_group and branched-chain amino acids (BCAAs)/4-pyridoxic acid may reflect microbiota-mediated nitrogen metabolism reprogramming and vitamin B6-dependent antioxidant pathways protecting sperm DNA integrity, consistent with reports of spermatogenic defects in B-vitamin-deficient mammalian models.

KEGG pathway enrichment analysis highlighted short-chain fatty acid metabolism, bile acid biosynthesis, and vitamin B6 metabolism as key pathways through which microbiota multidimensionally regulate semen quality via the “metabolite-host” axis: (1) Short-chain fatty acids act as energy substrates and epigenetic regulators to support the sperm mitochondrial function [61]; (2) Bile acid balance influences membrane stability and oxidative stress levels [62]; (3) Vitamin B6 metabolites maintain sperm genomic stability by scavenging ROS [63]. Gut microbiota participate in vitamin synthesis and metabolism, with B vitamins proven to regulate semen quality [64]. In practice, restoring vitamin-related gut microbiota or directly supplementing diets with appropriate vitamin levels may improve semen quality [65,66]. However, direct studies on gut microbiota-mediated vitamin metabolism and semen quality are limited, necessitating future research on optimal vitamin supplementation levels.

*Lactobacillus acidophilus* and *Lactobacillus* gasser utilize bile salt hydrolase (BSH) to hydrolyze primary bile acids into secondary bile acids, which influence glucose and lipid metabolism via farnesoid X receptor (FXR) and G protein-coupled bile acid receptor 1 (TGR5) pathways, leading to sperm abnormalities. Han et al. observed that hydroxytyrosol supplementation in Duroc boar diets increased *Coprococcus* abundance, reduced plasma bile acid levels, and promoted spermatogenesis [67]. Martinot et al. reported that taxifolin supplementation in Duroc boars decreased *Prevotella* abundance, lowered plasma bile acid concentrations, and reduced sperm deformity rates [68]. These studies highlighted the potential of regulating bile acid levels to improve semen quality. This multi-pathway synergy model provides a novel perspective on the gut–testis axis mechanism. This study identifies potential links between the abundance of specific microbial taxa and metabolites (e.g., SCFAs and PLA) and sperm quality through phenomics-based integrated analysis; yet, future validation in larger-scale cohorts remains necessary to confirm these relationships. In conclusion (Table 4), this study is the first to reveal that boar gut microbiota regulate semen quality through metabolite-mediated “gut-testis” crosstalk, offering a theoretical foundation for targeted microbiota modulation to enhance swine reproductive performance. Follow-up research should focus on probiotic/prebiotic interventions and explore key metabolites (e.g., butyrate, 4-pyridoxic acid) as biomarkers for reproductive health.

## 5. Conclusions

This study integrated 16S rRNA sequencing with untargeted metabolomics to reveal the potential regulatory relationship between the gut microbiota and metabolites of Tibetan boars and semen quality. The study indicates that the high semen utilization group (HJ) is dominated by Firmicutes and Proteobacteria, with higher community uniformity. The core bacterial genera (such as *Ruminococcus* and *Lactobacillus*) are associated with the enrichment of short chain fatty acids (butyric acid, valeric acid) and phenyllactic acid; The accumulation of lithocholic acid and corticosterone in the low utilization group (CJ) may impair sperm membrane stability. Based on this, the “Microbial Metabolite Semen Quality” interaction network theory is proposed, suggesting that pathways such as butyric acid metabolism may be related to the regulation of the gut testicular axis, providing a theoretical basis for probiotic intervention and metabolite supplementation strategies. These findings establish the potential role of gut microbiota in regulating male fertility, which will have practical value for improving livestock breeding efficiency.

## Figures and Tables

**Table 1:** Feed formulation and nutrient composition of diets.

Items	Proportion/%
Corn	45.00
Wheat bran	35.00
Soybean meal	16.00
Additive premixes 2624	4.00
DE (Digestible Energy Mcal/kg)	2.8487
CP (Crude Protein %)	15.89
CF (Crude Fat %)	3.94
Ca (Calcium %)	0.70
TP (Total Phosphorus %)	0.66
NPP (Non-Phytate Phosphorus %)	0.30
Lys (Lysine %)	0.79
Met + Cys (Methionine + Cystine %)	0.53
Thr (Threonine %)	0.58
Trp (Tryptophan %)	0.21

**Table 2:** The primer sequence information.

Target	Amplified Region	Primer Name	Primer Sequence (5′ → 3′)	Amplicon Length (bp)
16S Bacteria	V3-V4	341F	CCTACGGGNGGCWGCAG	~466
		806R	GGACTACHVGGGTATCTAAT	
16S Bacteria	V5-V7	799F	AACMGGATTAGATACCCKG	~414
		1193R	ACGTCATCCCCACCTTCC	
16S Archaea	V4-V5	Arch519F	CAGCMGCCGCGGTAA	~397
		Arch915R	GTGCTCCCCCGCCAATTCCT	
18S	V4	528F	GCGGTAATTCCAGCTCCAA	~179
		706R	AATCCRAGAATTTCACCTCT	
ITS	ITS2	ITS3_KYO2	GATGAAGAACGYAGYRAA	~381
		ITS4	TCCTCCGCTTATTGATATGC	
ITS	ITS1	ITS1_F_KYO2	TAGAGGAAGTAAAAGTCGTAA	~366
		ITS86R	TTCAAAGATTCGATGATTCAC	

**Table 3:** Semen quality parameters of boars in different semen utilization groups.

Semen Quality Parameter	High Utilization Group	Low Utilization Group	*p* Value
Semen utilization rate %	88.8025 ± 2.31535	73.2025 ± 18.62576	0.044
Sperm motility %	83.945 ± 1.69632	55.575 ± 24.74639	0.033
Sperm density 10^9^/ML	1.515 ± 0.07141	1.18 ± 0.55516	0.024
Progressive motility %	49.6425 ± 2.85477	42.2325 ± 13.48129	0.018

**Table 4:** Speculation on relevant critical paths and correlations.

Metabolite/Pathway	Mechanism	Associated Taxa
Butyrate	Energy provision/Anti-inflammation	*Faecalibacterium*, *Ruminococcus*
6-Hydroxyhexanoate	Xenobiotic detoxification/ATP synthesis	Streptococcaceae
Lithocholic acid (LCA)	Bile acid homeostasis/Membrane stability	*Clostridium* sensu stricto 3
Phenyllactic acid (PLA)	Antimicrobial/ROS scavenging	*Lactobacillus*
Vitamin B6 metabolism	DNA protection/Antioxidant	Rikenellaceae_RC9_gut_group

## Data Availability

The original contributions presented in this study are included in the article. Further inquiries can be directed to the corresponding authors.
